# Supplementary material for: The Impact of an Anchoring Layer on the Formation of Tethered Bilayer Lipid Membranes on Silver Substrates
Source: Molecules. 2021 Nov 15;26(22):6878. doi: 10.3390/molecules26226878 (PMC8624891; doi:10.3390/molecules26226878)
Supplement: Supplementary file 1 [file molecules-26-06878-s001.zip › molecules-1428579-supplementary.pdf]

Supporting Information

The Impact of Anchoring Layer on Formation of Tethered Bilayer Lipid Membranes on Silver Substrates

Indrė Aleknavičienė , Martynas Talaikis, Rima Budvytyte* and Gintaras Valincius

Experimental section

A biological relevance of tBLM on Silver Substrates containing different short-chain backfiller molecules

Despite the inferior initial electrical properties of tBLMs on SAMs (Figure 7) composed using longer backfillers (4M1B, 6M1H and 9M1N), the impact of VLY is still clearly visible in all EIS spectral changes (Figure S1 and S2, S3). Current data shows the functionality of tBLMs, using all spacers: 4M1B, 6M1H and 9M1N.

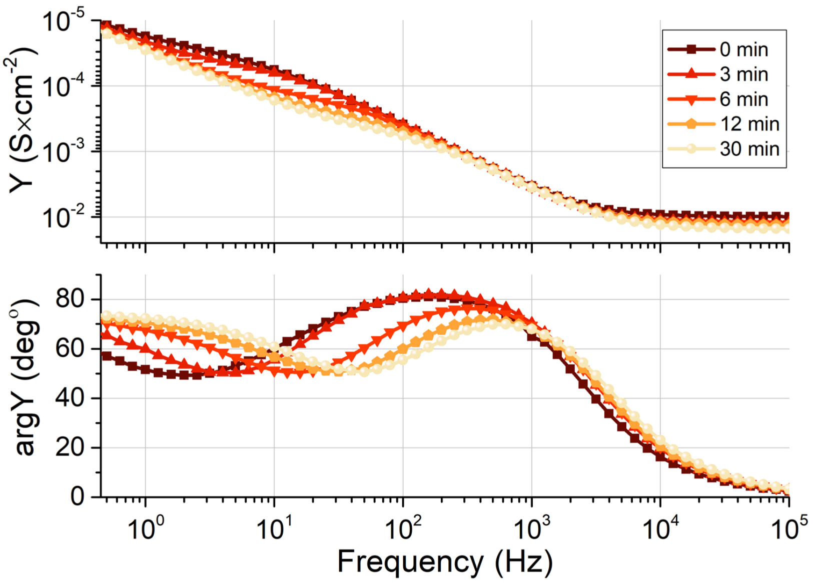

Figure S1. EIS spectral transformations of tBLMs upon exposure to VLY on time (initial, 3 min, 6 min, 12 min, 30 min). Admittance modulus (upper panel) and phase (lower panel) (Bode) plots.. 2nM VLY kinetics on tBLM were formed on mixed WC14/4M1P SAM and completed with DOPC/Chol (in ratio % 60:40) phospholipid mixture.

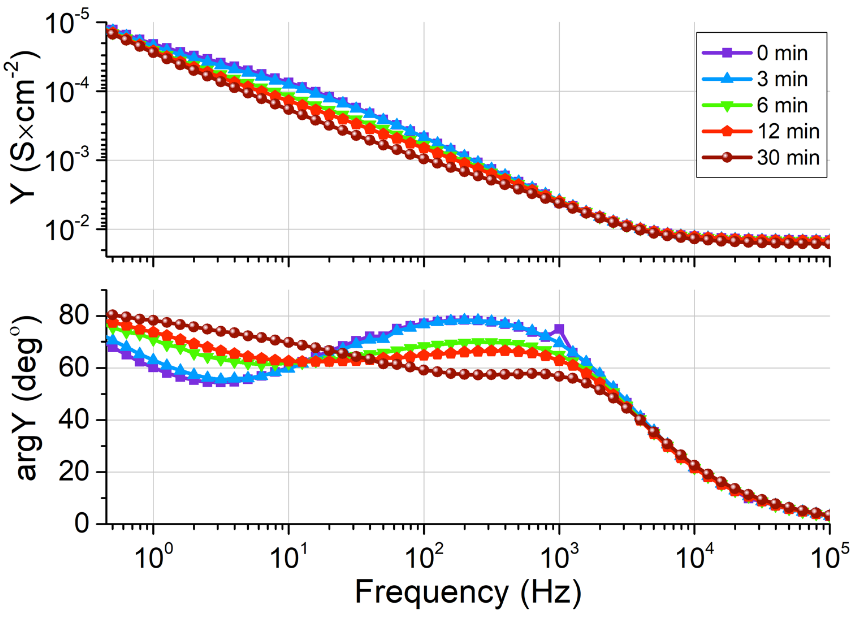

Figure S2. EIS spectral transformations of tBLMs upon exposure to VLY on time (initial, 3 min, 6 min, 12 min, 30 min). Admittance modulus (upper panel) and phase (lower panel) (Bode) plots.. 2nM VLY kinetics on tBLM were formed on mixed WC14/6M1P SAM and completed with DOPC/Chol (in ratio % 60:40) phospholipid mixture.

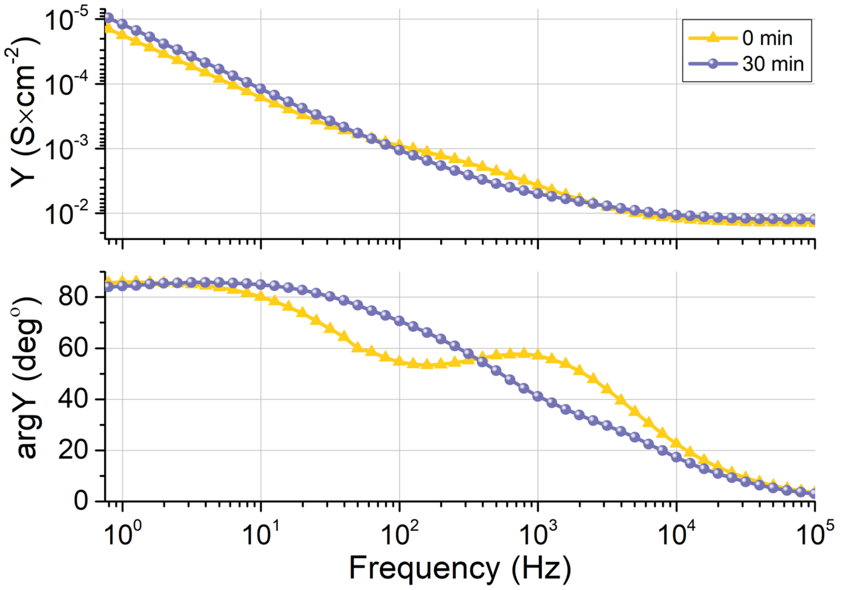

Figure S3. EIS spectral transformations of tBLMs upon exposure to VLY on time (initial and 30 min). Admittance modulus (upper panel) and phase (lower panel) (Bode) plots.. 2nM VLY kinetics on tBLM were formed on mixed WC14/9M1P SAM and completed with DOPC/Chol (in ratio % 60:40) phospholipid mixture.
